# Inferring Agronomical Insights for Wheat Canopy Using Image-Based Curve Fit *K*-Means Segmentation Algorithm and Statistical Analysis

**DOI:** 10.1155/2022/1875013

**Published:** 2022-01-31

**Authors:** Ankita Gupta, Lakhwinder Kaur, Gurmeet Kaur

**Affiliations:** ^1^Department of Computer Science and Engineering, Punjabi University, Patiala 147002, India; ^2^Department of Electronics and Communication Engineering, Punjabi University, Patiala 147002, India

## Abstract

Phenomics and chlorophyll fluorescence can help us to understand the various stresses a plant may undergo. In this research work, we observe the image-based morphological changes in the wheat canopy. These changes are monitored by capturing the maximum area of wheat canopy image that has maximum photosynthetic activity (chlorophyll fluorescence signals). The proposed algorithm presented here has three stages: (i) first, derivation of dynamic threshold value by curve fitting of data to eliminate the pixels of low-intensity value, (ii) second, extraction and segmentation of thresholded region by application of histogram-based *K*-means algorithm iteratively (this scheme of the algorithm is referred to as the curve fit *K*-means (CfitK-means) algorithm); and (iii) third, computation of 23 grey level cooccurrence matrix (GLCM) texture features (traits) from the wheat images has been done. These features help to do statistical analysis and infer agronomical insights. The analysis consists of correlation, factor, and agglomerative clustering to identify water stress indicators. A public repository of wheat canopy images was used that had normal and water stress response chlorophyll fluorescence images. The analysis of the feature dataset shows that all 23 features are proved fruitful in studying the changes in the shape and structure of wheat canopy due to water stress. The best segmentation algorithm was confirmed by doing exhaustive comparisons of seven segmentation algorithms. The comparisons showed that the best algorithm is CfitK-means as it has a maximum IoU score value of 95.75.

## 1. Introduction

Wheat is one of the essential staple food grains of the human race. As per Indian statistics, 80 percent of water is consumed by just three major crops—rice, wheat, and sugarcane. However, due to an exponential increase in the human population, its consumption has been increased to 10-fold [1]. At the same time, climatic changes are creating unexpected changes in the pattern of rains, leading to various kinds of abiotic stresses, predominantly in wheat crops. Due to these two main reasons, technological advancements are required to cope with such situations. The marker-assisted selection (MAS) method [2] has brought much confidence in current researchers to overcome the challenges of pest attacks and other stresses such as drought [3]. This technique primarily involves finding linkages between underline genes by identifying traits or modifying traits [4]. Most of these techniques are based on protein-based markers and require sophisticated spectrometry equipment [5]. However, certain traits can be understood with the help of three phenomena: reflectance, absorption, and emittance, that occur naturally in all plants [6]. The current record on the advancements in agriculture instrumentation shows that all these phenomena can be captured with the help of imaging sensors [7]. It has been found that the best way to quantify water stress in plants is by measuring the reemittance of light from the plant surface. Empirical observations show that when the plant changes its photochemistry, it emits a chlorophyll fluorescence signal which falls under the range of 680 nm to 800 nm as a by-product of photosynthesis [8]. These fluorescence wave signals are recorded with the help of imaging devices, where reemitted light can be realised as a digital image, and mathematical analysis 4can be done for quantification of photosynthetic activity and water stress in a nondestructive manner [9, 10].

Many researchers are utilizing high-resolution imagery [11] for doing germplasm evaluation [12], and various corroborating yields and trials have been done under different environments. The primary application includes observing physical damages. Satellite imagery is also helpful in predicting disease outbreaks in plants. A similar work can be done with UAVs and aerial vehicles such as parachutes, fixed-wing systems [13], rotocopters [14], and blimps [15]. The phenotyping of plants is also done with the help of ground-based vehicles fitted with an array of sensors for imaging crops at various stages of the lifecycle of crops [6]. It is a well-established fact that photosynthesis directly relates to the presence of water content in the plant material [16, 17]. In the next section, the various methods that help understand the interaction among the variables involved in photosynthesis activity, considering both normal and water scarcity conditions, have been discussed.

## 2. Review

Many plants undergo significant changes in the structure and development of their organs due to changes in environmental conditions [18]. The changes in the plant physiology in response to some stress or light conditions can be tracked using image processing methods [19, 20]. The images can capture chlorophyll concentration levels by capturing chlorophyll fluorescence signals. Hence, this current section discusses the various methods and procedures used for constructing Agronomical Insight Model (AIM) to deeply understand biological systems. These models in context to crop breeding domains help to investigate the behaviour of different plant response variables using various algorithms, which in turn attempt to identify the reasons due to which a particular event or a process occurred in a plant [21]. It involves two kinds of variables, the first category of variables are those variables due to which a particular phenomenon may occur, such variables are categorized as “cause variables,” and the second category of variables are those variables that are impacted due to these cause variables categorised as “effect variables.” Mathematically, “effect variables” is a kind of composite variable from which inferences can be drawn about the leading cause of a particular phenomenon, e.g., the plant's response under water scarcity. For deeper analysis and automation, many of the researchers are using either statistical analysis or machine learning as a way of finding data patterns and relationships among the variables [22].

Training manuals and books [23] in the context of modelling the behaviour of variables show that there are primarily four steps in designing the Agronomical Insight Model (AIM); in the first step, variables involved in phenomena are identified. In the second step, the ranking of the factors is done so that relevant and statistically significant variables can be sorted out. The third step is the classification or grouping of the factors involved in the phenomena. The last step is to find appropriate algorithms or equations for mapping various variables. This helps to find the impact of which variables a particular behaviour of variables changes in a system. In addition to these steps, many researchers are taking the help of domain experts or using a simulated model to understand the various interplays of the components of a system under observation. Many statisticians [24] further suggest that to identify correct contributing factors such as latent/hidden/internal variables and into a category known as “explicit variables” (a variable whose behaviour is already observable and is measurable).

In the second step for building an AIM model, many authors use different sorting algorithms to rank or find the importance of these variables. The current literature shows the most common methods used for this are importance ranking [25], Boruta analysis, factor analysis [26], principal component analysis [27], independent component analysis, correlation methods [28], and maximum likelihood method. All these methods do either an exploratory analysis of the variables or confirmatory factor analysis to find the behaviour and degree of association between the multiple variables, which in turn helps to establish grouping of the measured variables [29]. One of the popular methods used as an alternative to factor analysis is SEM (structural equation modelling) approach that helps to understand the plant response variables. Using these methods, the researchers build a consensus on how to group the cause-and-effect variables as per the plant's responses to the stimuli.

Oinam and Mehta [30] have done correlation, path, and regression analysis of the genomes variables of wheat bread variety. This study tries to understand the behaviour of genome traits under drought and normal irrigation conditions. Fouad have constructed ten regression models for finding out which trait can explain the behaviour of other traits under drought and normal conditions. Correlation analysis is conducted on various agronomical features utilized in the study, and it was found that grain weight directly affects the grain yield under irrigation and drought conditions [31]. A research work has claimed that a model consisting of three variables, which include “no. of grains per spike, no. of spike per plant, and hundred-gram weight” which are best predictors of yield; this model was identified by using stepwise regression. This argument has been further reinforced by the *R*^2^ value (0.93). In [32], Li et al. have worked on morphological and phenological trait classification of the wheat plant by using the concept of transfer learning to build the classifiers. The pretrained model used for this study is known as “ImageNet.” They trained the network as “Wheat Net.” The proposed system achieved a high level of accuracy (98%) for classifying the trait change that occurs moving from different stages (vegetative to flowering). A superpixel algorithm has been used for segmentation and developed the dataset of Tamura and GLCM features. These features were subjected to a support vector machine algorithm to resolve the three-class classification problem. They claimed an accuracy of 98.97% with the SVM classifier [33].

Boussakouran et al. worked on six different wheat varieties released between 1984 and 2007 [34]. All these varieties were grown under two kinds of water regimes: irrigated and rain-fed. Multiple morphological traits have been used for mapping the behaviour under these two irrigation conditions. The correlation analysis between morphological attributes such as green leaf area, spike area, spike length, flag leaf length, and flag leaf area has been performed. Later on, a stepwise regression analysis was applied to develop a stress susceptibility index. It was also found that the green leaf area was positively correlated to flag leaf length in the rain-fed area. However, there was a negative relationship between the grain filling period and days from sowing. Peymaninia et al. [35] have done a correlation analysis of morphological features of 12 bread wheat varieties genotypes. The main variables studied under this research work include biological yield, spike length, spike weight, number of grains per spike, fo, fm, fv, and fv/fm (ratio of fluorescence fv (variable fluorescence) to fm (maximum fluorescence)). Peymaninia et al. [35] found that spike length had the most positive direct effect on yield under drought conditions.

The paper [36] discusses the physiological and biochemical responses of the plant and underwater stress with the help of diagrams and illustrations. The most predominant factors impacted by the induced drought stress are relative water content (RWC), photosynthetic rate, and uptake of nutrients, which can be reflected by visually distinct changes in the plant's color and texture. From this review, it can be concluded that most researchers' primary focus is to build models that would help them predict or map the yield of a particular crop. Little work has been reported in context to understanding the behaviour of variables among themselves irrespective of yield. Multiple studies track morphological features, physiological features, features related to gaseous states, photosynthetic activity rate under water stress, and controlled conditions. The traits are tracked using different imaging modalities, i.e., thermal imaging [37] infrared imaging, visible-light imaging, tomography, NIR (near-infrared) imaging, and chlorophyll fluorescence imaging [38]. Research also gives evidence that the leaf area, grain size, no. of grains per spike, spike length, etc. are impacted due to water stress, and at the same time, these variables, when tracked through image processing techniques, are required to be mapped with mathematical functions. These mathematical functions compute either statistical values or values that are computed with an algorithm such as Tamura and GLCM features [39].

In the summary of the literature review, it can be said that little work has been found where the plant traits extracted from images are mathematically modelled. In this research work, an attempt has been made to understand the various aspects of wheat canopy changes when there is a stress stimulus such as water stress.

## 3. Materials and Methods

This research initiative is aimed at analysing image-based indicators of wheat plants that have some agronomical relationship to draw inferences among them. These indicators are traits in an agronomic sense that indicates the reaction of the plant under water stress. For this reason, a publicly available image dataset has been used as it has instances of water-stressed and controlled wheat plant images [40]. The dataset contains chlorophyll fluorescence images of Raj 3765 varieties of wheat [41]. There are a set of 24 images each (control and drought) experiment captured for 60 days. RGB image dataset having (720 × 2) instances of both control and drought with the resolution of 72 dpi. After feature extraction, a feature dataset was created and deposited with the public repository to maintain the reproducibility of this work. The dataset and code used for building a statistic model are publicly available [42, 43]. The images were captured using an indoor laboratory facility with the help of a chlorophyll fluorescence-based camera. Most of the images in this dataset do not require additional transformation except that the images need to have the same aspect ratio and the same size (1388 (width) × 1038 (frequency)).

### 3.1. Proposed Technique

In the context of this research, color features, texture, and shape features are important for understanding the agronomic implications of CF images. Preliminary color analysis of the images shows that all the three channels of the image have almost similar intensity levels at respective timelines of PSII activity. Since the amount of photosynthesis activity is dependent upon how we interpret the pixel values, it will not be prudent to track just color intensities. As color analysis only gives information concerning the frequency and wavelength of the light passing through the plant body. Texture analysis through experimentation proved a better indicator of morphological or shape changes in the wheat canopy as compared to color analysis.

**Figure 1:**
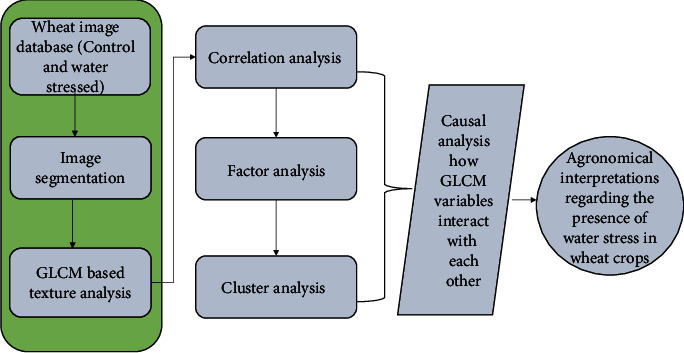
Flow chart of study methodology.

Hence, the texture features will be extracted after the pre-processing (thresholding and segmentation of the wheat canopy images). Since the dataset will have texture features extracted for both control and water-stressed wheat plants, the relationship among the GLCM variables will represent both conditions. The GLCM features of each image were extracted by using a GLCM matrix of 9∗9 and processed with the quantization level of 3. The relationships between the variables are identified using correlation, factor analysis, and clustering analysis methods [44]. The implementation process has been shown in (see Figure 1).

**Figure 2:**
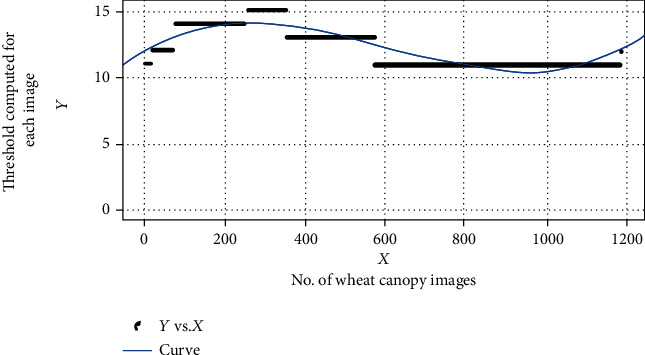
Output of curve fitting mathematical function.

The proposed technique works in the following three phases:

1. Phase 1: segmentation—new curve fit *K*-means segmentation technique has been proposed (see Figure 2)

(2) Phase 2: feature extraction—GLCM texture features are extracted from the segmented image
(3) Phase 3: Agronomical Insight Modelling (AIM) was done using correlation, factor, and cluster analysis to identify the variables that hang together for water stress identification

#### 3.1.1. Segmentation of Wheat Canopy

In the context of our research work, predominantly few images require some contrast enhancement, removal of artefacts, RGB to grayscale transformation, and aspect ratio correction. As per the problem undertaken here, the dataset consists of the traits or features that can indicate a change in the wheat canopy when water stress influence the growth of the wheat plant [45]. To extract such traits [46], seven segmentation algorithms were employed. Later on, the output was put under a rigorous validation process for assuring that the best clustering-based segmentation algorithm is constructed for wheat canopy segmentation.

The current literature has copious shreds of evidence in making most of the segmentation algorithms automatic; it is necessary to select the threshold “*T*” automatically. For this, researchers have employed multiple ways to compute the intensity threshold for their algorithms. Few authors have used the histogram shape, where the peaks, valleys, and intervals of the histogram provide hints for finding the thresholds to segment the objects embodied in the image [32]. Other sets of researchers have used clustering-based methods, in which the grey-level samples are clustered in two parts as background and foreground (object), or alternately modelled as a mixture of two Gaussians. Then, entropy-based methods use the entropy of the foreground and background regions to segment objects; furthermore, the cross-entropy between the original and binarised image is computed for reliable segmentation. Object attribute-based segmentation methods search for a measure of similarity between the grey-level and the binarised image pixels for realising the segments, fuzzy shape similarity, and edge cross-intersect [5, 6]. Few researchers have worked on spatial methods. These methods use a higher-order probability distribution or correlation between pixels to segment the objects. Local methods adapt the threshold value (*T*) for each pixel to the local image characteristics. A different *T* is selected for each pixel block in an image in these methods.

Further, data from the segmentation experiments reveal that the automatic threshold works best when an excellent background to foreground contrast ratio exists; i.e., image is captured under good lightening conditions with minimal noise. In the context of the problem undertaken and from the initial analysis, it becomes clear that the difference between the foreground and background is negligible. The difference further gets reduced to a minimal value when initially Global Static Thresholding (GST) is applied. By considering these facts, each image was passed through Global Static Thresholding (GST), where threshold value was computed using linear curve fitting as shown in Equation (1), followed by the *K*-means segmentation algorithm, feature extraction, and feature analysis. The various steps used in the proposed technique are as follows:

#### 3.1.2. Step 1: Threshold Calculation

The visual inspection of the CF (chlorophyll fluorescence) image dataset shows that there are possibly three types of clusters that can be found in the CF images. The first one is the background (pixel intensity = = 0), the second is the wheat canopy, and the third one is the pot in which the wheat plant is grown. Hence, to extract only that part of the image that represents “photosynthetic activity,” there is a need to remove the pot in which the wheat plant is grown. The histogram analysis of the images as per the timeline shows that initially the pixels have low intensity, and by the end of the timeline, pixel intensity increases and in between in some cases, the pot is visible as in the images. The appearance of the pot is more in the case of the water-stressed images. Using the hit and trial method, it was found that elimination of unwanted pixels can be done using a dynamic range (11 to 17). In this way, reconstruction of the image represents a photosynthetic activity that can be done without those pixels. When pixels below 17 are removed, they also helped in removing the pot in which the wheat plant had been grown by allowing us to focus on the canopy itself for efficient analysis. To automate the process, an unsupervised clustering algorithm was found to be the best, provided the algorithm knows how to eliminate and cluster the image into foreground and background. Hence, the algorithm has two phases. In the first phase, removal of unwanted pixels is done using a segmentation algorithm that computes the pixel elimination threshold using the three-degree curve fitting method as shown in (see Figure 2), a mathematical function is constructed using

(1) $$\begin{array}{c} \text{Threshold}\left(x\right)=p_{1}x^{3}+p_{2}x^{2}+p_{3}x+p_{4}, \end{array}$$

where *p*_1_ = 2.273*e* − 08 (2.142*e* − 08, 2.403*e* − 08), *p*_2_ = −4.118*e* − 05 (−4.353*e* − 05, −3.882*e* − 05), *p*_3_ = 0.01699 (0.01579, 0.0182 ), and *p*_4_ = 11.95 (11.79, 12.12) are coefficients, with 95% of confidence bounds and *x* is an independent variable (threshold values found using the hit and trial method). The mathematical expression has been derived using the dataset consisting of 1188 records of thresholds values found using the hit and trial method. The selection of the mathematical equation has been done when SSE (sum of square error = 622.1, the *R*^2^ value was 0.956, the adjusted *R*^2^ was 0.9554, and the RMSE value was 0.7249).

In the second phase, maximization of the photosynthetic area is done. After thresholding *K*-means clustering algorithm with *K* = 2 (i.e., background and wheat canopy clusters) has been applied iteratively for clustering. For the clustering process, at the same time, compute the initial means/centroids by using values from the histogram analysis.

(2) $$\begin{array}{c} \left[\text{Histogram}\_\text{means}\right]=\text{mean }\left(\text{histogram}\left(\left[\text{data}>\text{cutoff}\right],\text{bin}=255\right)\right), \end{array}$$

(3) $$\begin{array}{c} \left[\text{intial}\_\text{mean}0\right]=\text{mean}\left(\text{histogram}\_\text{means}\right), \end{array}$$

(4) $$\begin{array}{c} \left[\text{intial}\_\text{mean}1\right]=\min\left(\text{histogram}\_\text{means}\right). \end{array}$$

The algorithm does not randomly choose centroids; the centroids are provided using Equations (2)–(4). This is the *K*-means working process which starts iterations with intelligent guesses and then assigns each observation to its closest centroid, based on the Euclidean distance between the pixel and the centroid. For each of the *K* clusters (in our case, it is fixed at 2), update the cluster centroid by calculating the new mean values of all the data points in the cluster. The centroid of a *K*^th^ cluster is a vector of length “*p*” containing the means of all variables for the observations in the *K*th cluster; *p* is the number of variables. Iteratively minimize the total within the sum of squares. The images are normalized before the execution of the algorithm is done. That is, iterate steps 3 and 4 until the cluster assignments stop changing or the maximum number of iterations is reached.

The pseudocode of the *K*-means clustering algorithm is as follows:

1. Specify the number of clusters *K* = 2 and iterations = 20
2. Initialize centroids by first shuffling the dataset and then randomly selecting *K* data points for the centroids without replacement
3. Keep iterating steps (iii) to (vi) until there is no change to the centroids; i.e., the assignment of data points to clusters is not changing
4. Compute the sum of the squared distance between data points and all centroids
5. Assign each data point to the closest cluster (centroid)
6. Compute the centroids for the clusters by taking the average of all data points that belong to each cluster. After getting the optimum canopy region, it is passed to the feature extraction algorithm

### 3.2. Segmentation Results and Discussion

**Figure 3:**
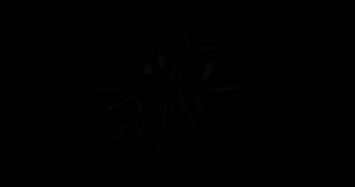
Segmentation algorithm used: none (input image from which wheat canopy will be extracted); description: original image to be segmented.

**Figure 4:**
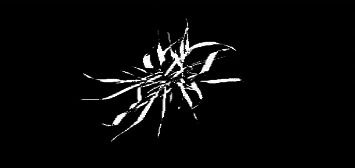
Segmentation algorithm used: Global Static Thresholding (fixed value); description: some pixel values lost membership in the final segmented image.

**Figure 5:**
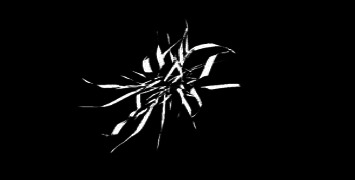
Segmentation algorithm used: Global Automatic Thresholding (Otsu); description: pixel membership loss is there but less than that of the static thresholding method.

**Figure 6:**
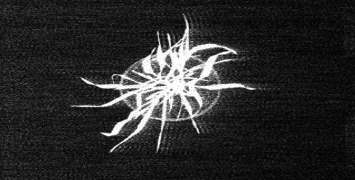
Segmentation algorithm used: *K*-means based on 4 mean values (Otsu); description: pixel membership loss is there but less than that of the static thresholding method.

**Figure 7:**
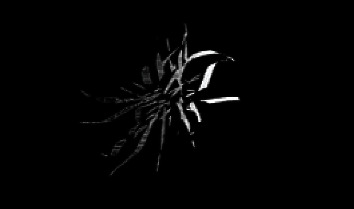
Segmentation algorithm used: watershed; description: the watershed algorithm failed to identify the boundaries properly.

**Figure 8:**
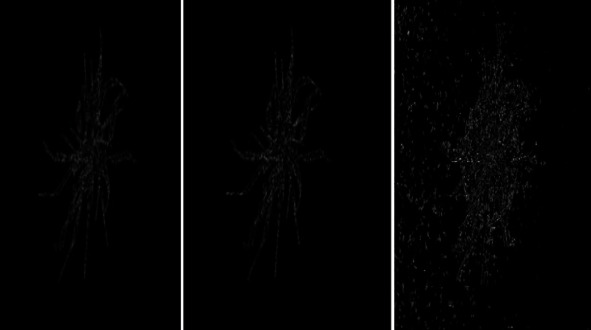
Segmentation algorithm used: convolution gradient filters (Sobel, Prewitt, Canny); description: nonsmooth edges for all the three operators.

**Figure 9:**
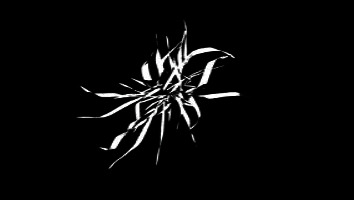
Segmentation algorithm used: CfitK-means; description: best results.

For qualitative comparison of the proposed methodology, the segmentation results on the input image (see Figure 3) using seven segmentation algorithms, namely, Global Static Thresholding (GST) (see Figure 4), Global Automatic Thresholding (GAT) (see Figure 5), *K*-means based on four mean values (see Figure 6), Watershed (see Figure 7), mean shift (overhead of running mean shift is high and does not give significant output), convolution gradient filters (see Figure 8), and curve fit‐based thresholding + *K*‐means algorithm named as CFitK-means (see Figure 9) have been shown. A closer look at the output images demonstrates the superiority of the proposed CFitK-means method as compared to other methods (see Figure 9).

To analyse the accuracy of the proposed segmentation process, a random sampling method has been applied and mutually exclusive sets of images were created. The minimum sample size taken was 25 and the maximum was 100.

1. The ground truth or valid segmented images were decided by visual inspection and a separate set of such images was created for running evaluation tests. The intersection (IoU) between the target (valid segmented image) and predicted (segmented image by different algorithms) was computed using the following equation:

(5) $$\begin{array}{c} \text{IoU score}=\frac{\text{target}\cap \text{prediction}}{\text{target}\cup \text{prediction}} \end{array}$$

(2) The IoU values are computed using different sample sizes, and average values for each sample evaluation round were computed.  Table 1 gives the IoU values computed using Equation (5) for all the algorithms that are competing with each other in terms of accuracy for wheat canopy extraction. The results of the mean shift, watershed, Sobel, canny, and Prewitt algorithms were ignored, as visually it was abundantly clear that the segmented output was not satisfactory. An average IoU score above 0.5 cut-offs was considered an acceptable result for completing the segmentation process. Four evaluations were done against the ground truth/valid segmented image dataset as shown in (Table 1)

**Figure 10:**
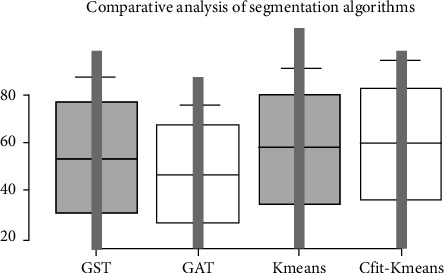
IoU score comparison for performance.

It can be observed from the boxplot (see Figure 10) that the CFitK-means algorithm has the maximum IoU score. This means that this hybrid segmentation algorithm gives a maximum number of valid segmented images as per the ground truth set of images. In each mutually exclusive random sample of evaluations, the number of correctly segmented images is highest for this segmented algorithm.

### 3.3. Image-Based Phenomics Trait Extraction

The current literature [47, 48] gives many pieces of documentary evidence that the texture features of images can be represented as characteristics of the objects embodied in the images. Many researchers point out that texture features can provide better information as compared to color features. In this research work, the GLCM features aided us to extract information on the variations of the morphology that occur when the plant is responding to stress or stimuli. Therefore, by using the GLCM algorithm on the canopy images obtained from the previous step, twenty-three features were extracted and 1188 instances were created. The description of all these features is given in (Table 2).

*P*(*i*, *j*) is the cooccurrence matrix, and *N*_*g*_ is the no. of grey levels.

(6) $$\begin{array}{c} p\left(i,j\right)=\frac{P\left(i,j\right)}{\sum P\left(i,j\right)}\text{ is the normalized cooccurrence matrix}, \end{array}$$

(7) $$\begin{array}{c} p_{x}\left(i\right)=\overset{N_{g}}{\underset{j=1}{\sum }}P\left(i,j\right)\text{are the marginal row probabilities}, \end{array}$$

(8) $$\begin{array}{c} p_{y}\left(i\right)=\overset{N_{g}}{\underset{i=1}{\sum }}P\left(i,j\right)\text{are the marginal column probabilities}, \end{array}$$

(9) $$\begin{array}{c} \mu_{x}=\overset{N_{g}}{\underset{j=1}{\sum }}p\left(i,j\right)\text{ is the mean grey level intensity of }p_{x}, \end{array}$$

(10) $$\begin{array}{c} \mu_{y}=\overset{N_{g}}{\underset{i=1}{\sum }}p\left(i,j\right)\text{is the mean grey level intensity of }p_{y}, \end{array}$$

(11) $$\begin{array}{c} \sigma_{x}^{2}=\overset{N_{g}}{\underset{i=1}{\sum }}{\left(i-\mu_{x}\right)}^{2}p_{x}\left(i\right)\text{is the variance of }p_{x}, \end{array}$$

(12) $$\begin{array}{c} \sigma_{y}^{2}=\overset{N_{g}}{\underset{j=1}{\sum }}{\left(i-\mu_{y}\right)}^{2}p_{y}\left(j\right)\text{is the variance of }p_{y}, \end{array}$$

(13) $$\begin{array}{c} p_{x+y}\left(k\right)=\overset{N_{g}}{\underset{i=1}{\sum }}\overset{N_{g}}{\underset{j=1}{\sum }}p\left(i+j\right),\text{where }i+j=k,k=2,3,\cdots ,2\;N_{g}, \end{array}$$

(14) $$\begin{array}{c} p_{x-y}\left(k\right)=\overset{N_{g}}{\underset{i=1}{\sum }}\overset{N_{g}}{\underset{j=1}{\sum }}p\left(i-j\right),\text{where }\left|\;i–j\;\right|=k,k=0,1,\cdots ,N_{g}–1, \end{array}$$

(15) $$\begin{array}{c} \mu_{x+y}=\overset{2N_{g}}{\underset{k=2}{\sum }}k.p_{x+y}\left(k\right), \end{array}$$

(16) $$\begin{array}{c} \mu_{x+y}=\overset{2N_{g}}{\underset{k=2}{\sum }}k.p_{x+y}\left(k\right), \end{array}$$

(17) $$\begin{array}{c} \text{HX}=-\overset{N_{g}}{\underset{i=1}{\sum }}p_{x}\left(i\right)\log_{2}\left(p_{x}\left(i\right)+\in \right), \end{array}$$

(18) $$\begin{array}{c} \text{HY}=-\overset{N_{g}}{\underset{j=1}{\sum }}p_{y}\left(j\right)\log_{2}\left(p_{y}\left(j\right)+\in \right), \end{array}$$

(19) $$\begin{array}{c} \text{HXY}=-\overset{N_{g}}{\underset{i=1}{\sum }}\overset{N_{g}}{\underset{j=1}{\sum }}p\left(i,j\right)\log_{2}\left(p\left(i,j\right)+\in \right), \end{array}$$

(20) $$\begin{array}{c} \text{HXY}1=-\overset{N_{g}}{\underset{i=1}{\sum }}\overset{N_{g}}{\underset{j=1}{\sum }}p\left(i,j\right)\log_{2}\left(p_{x}\left(i\right)p_{y}\left(j\right)+\in \right), \end{array}$$

(21) $$\begin{array}{c} \text{HXY}2=-\overset{N_{g}}{\underset{i=1}{\sum }}\overset{N_{g}}{\underset{j=1}{\sum }}p_{x}\left(i\right)p_{y}\left(j\right)\log_{2}\left(p_{x}\left(i\right)p_{y}\left(j\right)+\in \right). \end{array}$$

Equations (7)–(21) give mathematical explanations of the notations used in (Table 2). Further for obtaining agronomical inferences and insights from image-based metrics, multiple statistical methods are explained in the coming sections. To find which image-based metrics can give a crystal-clear indication of water stress correlation analysis, factor analysis and cluster analysis are given. The correlation analysis will help us to eliminate those variables that do not give any hint about agronomical structural variation in the wheat canopy. The factor analysis will identify “groups of variables” that give statistically significant evidence of changes in plants due to stress. For a deeper understanding of the behaviour of the groups and validation of the groups identified, cluster analysis will also be done.

### 3.4. Understanding the Image-Based Traits of Wheat Canopy

For building Agronomical Insight Models (AIM) models for wheat water stress identification, in this work, correlation analysis, cluster, and factor analysis have been used for the exploratory study of all the variables. Applying the factor loading analysis method can only be done if the dataset is suitable for it. Hence, first sampling adequacy was checked with the help of the Kaiser-Meyer-Olkin (KMO) test. It was found that the KMO value was 0.86, and from its value, it can be interpreted that sampling is adequate. The following method to find the suitability of the data was Bartlett's test of sphericity. This statistical test checks the premise that an identity matrix is the correlation matrix of the data exhibits and that they are sufficiently unrelated, thus unsuitable for structure detection. In this test, however, our dataset failed, and it was also found that some eigenvalues are also coming negative. Hence, the process of factor analysis was dropped for further analysis, and clustering analysis was adopted. The clustering method and its outcomes are explained in the next section. Hence, in this section, an attempt has been made to apply (i) correlation analysis, (ii) factor, and (iii) cluster analysis to map the variable's behaviour to find variables that “hang together” (i.e., have a strong association with each other). In terms of agronomical sense, it will help us find variables that respond somehow when there is stress or stimuli.

### 3.5. Correlation Analysis of Wheat Canopy Traits

The correlation analysis is a process that helps to identify all the pairs of variables/traits that hold some degree of association with other variables. A summary of the results obtained from all three methods (Spearman, Kendall, and Pearson) is shown in Table 3. For maintaining the readability of the paper, repetitive information regarding values of different correlation methods is omitted. The table categorizes the variables based on their correlation ranges. Since correlation gives an idea of how strong and weak the relationship is between the variables, a lower correlation value implies that the two variables slightly impact each other.

A change in one variable does not impact the values of other variables numerically. Higher values of correlation imply that variables are firmly attached to others. From (Table 3), it has been observed that variables such as “autoc,” “contr,” “cprom,” “cshad,” “dissi,” “energy, “entro,” “homom,” “homop,” “maxpr,” “sosvh,” “savgh,” “svarh,” “senth,” “dvarh,” “denth,” “homom.1,” and “indnc” are in the medium and high correlation categories. This means that all these twenty variables have some degree of association and impact each other directly or indirectly.

### 3.6. Factor Loading Analysis of Wheat Plant Traits

The application of factor analysis methods can only be made if the dataset is suitable. Hence, first sampling adequacy was checked with the help of the Kaiser-Meyer-Olkin (KMO) test. It was found that the KMO value was 0.86, and from its value, it can be interpreted that sampling is adequate. The method used to find the suitability of the data was Bartlett's test of sphericity. This statistical test checks the premise that an identity matrix is the correlation matrix of the data exhibits and that they are sufficiently unrelated, thus unsuitable for structure detection. In this test, however, our dataset failed, and it was also found that some eigenvalues are also coming negative. Hence, the process of factor analysis was dropped for further analysis, and clustering analysis was adopted. The clustering method and its outcomes are explained in the next section.

### 3.7. Clustering Analysis of Wheat Plant Traits

With the help of agglomerative clustering as shown in (Table 4), an attempt has been made to find a group of variables with some linkages as shown in (Table 5). Linkage implies that there is some homogeneity between variables in question. An association between multiple variables (clusters/groups) represents phenomena associated with specific plant behaviour abstracted from image-based features.

Our research work uses GLCM texture metrics to represent plant behaviour under water stress and normal conditions.  Table 4 is a numerical summary of the steps taken by the clustering algorithm for building clusters. It displays information regarding “stage no.” which tells the progress as the iteration occurs; “Cluster combined” as the iteration process passes through different stages the clusters get reorganised; this information is also available in Table 4, and “coefficients” are the value coefficients indicating the degree of similarity between the clusters. It is an iterative bottom-up process of finding clusters or groups.

The algorithm implemented here in the problem undertaken uses the nearest neighbour method to find similar data points/attributes/features. The dataset was first normalised to maintain the uniform scale of values. The coefficient column indicates the distance between the two clusters (or cases) joined at each stage. The distance is computed using the squared Euclidean distance formula. The values of coefficients depend on the proximity measure and linkage method used in the cluster analysis. It can be observed from the agglomeration schedule that the values of the coefficients remain relatively small until stage 18 as given in (Table 4), which implies that the similarity between the clusters formed is relatively high.

However, from stage 18, the similarity coefficients start changing to higher values till the values reach 257.481, which is stage 18. After this stage, there is substantial change till the coefficient value reaches 443. In the next stage, it can be observed that the coefficient value becomes huge (2099). From (Table 4), it can further be observed that there are three stages at which the algorithms have taken a chance to merge into three different clusters. When clusters are joined, they are subsequently given membership based on the minimum value of the two in terms of distance computed by algorithms as shown in (Table 5). To validate the membership of the variables concerning different clusters/groups, different distance measures and clustering methods were applied to find the stability of the solution, and the following output was realised based on the data points/attributes/features.

Table 5 gives the outcome of the clustering process. It can be observed from Table 5 that it has formed three groups of indicators. The selection of the three groups was based on prior knowledge about the nature of GLCM metrics. The prior information was derived from the mathematical expressions of GLCM metrics (indicators) and from the correlation matrix constructed in the previous step to decide the number of groups/clusters that are three.

**Figure 11:**
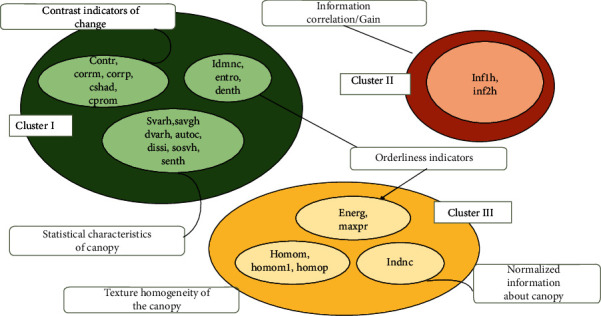
Visualization of clusters formed.

As shown in Figure 11, the Venn diagram is based on the outcome of the agglomerative clustering (single solution) using the Nearest Neighbour method with normalised data. The Nearest Neighbour method uses squared Euclidean distance for computing distances. Other methods (between groups linkage, within groups linkage, centroid clustering, furthest neighbour, median clustering, Ward's method) were also employed to validate the clustering membership outcome from this method. This was found that all the methods gave a similar output.

#### 3.7.1. Profiling of Clusters for Trait Analysis

In this section, we profile the clusters in two ways: first, it was at a level of GLCM metrics, and secondly, inferences have been derived from the cluster analysis that has agronomical application.

*(1) Cluster I*. This is the biggest cluster out of the three. The clustering process has found fifteen GLCM metrics hanging together, namely, “autoc,” “contr,” “corrm,” “corrp,” “cprom,” “cshad,” “dissi,” “entro,” “sosvh,” “savgh,” “senth,” “dvarth,” “denth,” “idmnc,” and “svarh.” It can be observed from their definition that most of these descriptors involve variance-based computations. The value of the metrics, contrast (local variations), and cluster-shade (skewness) indicate the texture present in the image. The cprom descriptor hints at the degree of symmetry in the image matrix. The change in the image's skewness value indicates that the image's symmetry gets impacted. This is also true; i.e., when the symmetry in the image changes the skewness also changes. This implies that whenever a slight change is induced in the wheat canopy due to some stimuli or stress; these variables will significantly change. The GLCM variables “sosvh” and “svarh” give information on changes that occur in terms of mean and variance. Since these variables have a strong relationship with each other according to correlation and factor analysis, it can be safely inferred that both these variables hang together. The variable “savgh” indicates the dispersion of pixels, which again gets impacted due to changes in mean and variance. The variables “autoc” indicates the consistency of coarseness in a pattern. It appears that changes in skewness and shift in symmetry create rapid changes in the coarseness of the image matrix. Mathematically, it is clear that if there are changes in the intensity of pixels and shifts in pixel positions (skewness and symmetry), the variance of pixel intensity also varies. Based on these facts, 15 variables/indicators have been identified that “hang together” or “change together” whenever there is a change in wheat plant canopy characterisation.

*(2) Cluster II*. Two GLCM metrics “inf1he” and “inf2h” form the third cluster. These GLCM metrics compute the dependency between the X and Y pixels by using probability, correlation, and information theory. The agglomeration algorithms seem to give membership to both these metrics in the same group because both these metrics give a similar type of information.

In summary, it can be observed that all of the 23 GLCM metrics give hints about different changes that are occurring in the wheat canopy. GLCM metrics can be grouped into three types, namely, “contrast group” (contrast, cluster shade, and cluster prominence), “Orderliness” (entropy and energy), and the last group consisting of metrics that give statistical characteristics and descriptions about the texture. Using GLCM metrics, many types of trait analysis of the wheat canopy can be done, and inferences can be drawn. This work also helps in bringing clarity and control in understanding the changes the wheat plant undergoes during the vegetative growth stage due to water stress (as the database consists of 594 instances of water stress also). This understanding helps to improve the quality and quantity of the breed by taking corrective steps.

*(3) Cluster III*. Cluster III has six GLCM metrics viz., “homomp,” “homom,” “maxpr,” “energy,” “homom1,” and “indnc.” This implies that two types of homogeneity metrics have a good deal of association linkage, and most likely, they convey similar information (uniformity in pixels) for analysis undertaken. The “maximum probability” metric has also found itself grouped in this cluster. This metric value is the largest entry (grey level) that corresponds to the most decisive response in the image. Energy metrics in terms of GLCM convey an amount of uniformity/homogeneity in the image. If a homogenous image contains few dominant grey shades, then the matrix will have few large entries. This confirms our hypothesis that image-based features can also be used for deriving agronomical inferences.

### 3.8. Agronomical Interpretations

In this subsection, a series of assertions are made to rationalise the outcomes by linking wheat plant behaviour with digital image processing which has been done [53, 54] (e.g., chlorophyll fluorescence images). The wheat plant's development features can be inferred from the characteristics of its canopy. Using the GLCM feature extraction process (texture analysis), we can track the appearance of the canopy that covers normal and water stress conditions.

1. Mathematically, it is clear that there are changes in the intensity of pixels, shift in pixel's positions (skewness and symmetry), and pixel intensity varies. Due to this fact, the 23 texture variables “hang together” or “change together” whenever a wheat plant canopy changes its stress response
2. Leaf shape helps to characterise the health of the wheat plant. The texture features that help to identify symmetry (cshad, cprom, and homogeneity) of the image can also help characterise the wheat plant's health in our case
3. Leaf size, area, density, and canopy is a collection of leaves and branches. The texture features that can track the size of the objects/pixel group contained in the image are helpful in this case. The glcm metric that tells us about the fineness or coarseness and homogeneity can help us hint about the change in the size of the leaf due to some stress. If a plant is under stress, leaf size will get reduced, and consequently, the leaf area and density will also reduce. A large leaf area implies a complex texture of leaves. GLCM features that track the dispersion of pixels which in turn helps to check the health of the wheat plant. The “savgh,” “sosvh,” “svarh,” and “glcm” metrics help to find indications of stress in the plant by tracking variance from the mean. Higher levels of dispersion of pixels higher will be the plant under water stress

## 4. Discussion

In summary, it can be observed that all of the 23 GLCM metrics give hints about different changes that are occurring in the wheat plant physiology. In the context of stress analysis, GLCM metrics can be grouped into three types, namely, “contrast group” (contrast, cluster shade, and cluster prominence), “Orderliness” (entropy and energy), and the last group consisting of metrics that give statistical characteristics and description about the texture. This research gives affirmation that by using GLCM metrics a lot of trait analysis of wheat canopy can be done easily and reliable agronomical inferences can be drawn. This work also helps to clarify and control the changes the wheat plant undergoes during the vegetative growth stage due to water stress.

## 5. Limitations and Conclusion

In this research, three methods (correlation analysis, factor analysis, and clustering analysis) were employed to understand the behaviour of plant response variables. The values of variables used in wheat research work were computed from the images of wheat plant canopy that had captured maximum photosynthetic activity. First, the performance of the number of segmentation algorithms has been analysed for wheat plant canopy segmentation. It was found that the curve fit-based *K*-means method is the most effective segmentation algorithm as it yields the highest IoU score (95.75%). Further, GLCM features were computed, followed by correlation, factor, and cluster analysis. From the analysis, it can be concluded with confidence that there are multiple groups of variables/traits that “hang together.” These indirect methods help quantify changes in the wheat plant physiology that can help us to avoid destructive methods to map the behaviour of a plant. It is clear from this research work that changes in the plant morphology due to changes in stimuli and stresses can be observed by measuring the values of mainly 23 texture variables. This statement is expressed based on the outcome of the clustering process and correlation analysis. This work is limited to a single wheat variety; a larger dataset with multiple wheat varieties is always desired. Large calibrated samples are required for the success of such experiments so that generalisations from the study are more accurate. Additionally, analysis based on other color models could have increased the robustness of the results for deeper agronomical insights.

## Figures and Tables

**Table 1:** Performance evaluation of segmentation algorithms in terms of IoU (intersection over union) score.

Algorithm	Sample size				Average IoU score
	25	50	75	100	
Global Static Thresholding (GST)	20	40	67	88	84.3
Global Automatic Thresholding (GAT)	19	33	60	76	74.5
*K*-means clustering	22	46	70	92	91.3
CFitK-means	24	48	72	95	95.75

**Table 2:** Description of glcm metrics that define wheat canopy morphology.

GLCM features	Formulae used	Agronomical implications
Autocorrelation (autoc): [49–52]	$\overset{N_{g}}{\underset{i=1}{\sum }}\overset{N_{g}}{\underset{j=1}{\sum }}\left(i,j\right)p\left(i,j\right)$	It helps to find how consistent the pattern is in an image matrix in terms of coarseness. Its value ranges from 0 to 1, and 1 conveys maximum coarseness. If there is high coarseness this means the canopy structure is having less density.

Contrast: (contr) [49–52]	$\overset{N_{g}}{\underset{i=1}{\sum }}\overset{N_{g}}{\underset{j=1}{\sum }}{\left(i-j\right)}^{2}p\left(i,j\right)$	This indicates the difference between the highest and lowest intensity pixels in an image. High contrast means that there is a huge difference between different parts of the object which will aid as a useful tool for canopy segmentation.

Correlation (corrm): [49–52]	$\frac{\sum_{i}^{N_{g}}\sum_{j}^{N_{g}}\left(i-\mu \right)\left(j-\mu \right)p\left(i,j\right)}{\sigma^{2}}$	Measures the joint probability of pairs of pixels under observation. This means the pixels within the structure of the canopy have some kind of association with each other in case there is some kind of correlation.

Correlation (corrp): [49–52]	$\frac{\sum_{i}^{N_{g}}\sum_{j}^{N_{g}}\left(i,j\right)p\left(i,j\right)-\mu^{2}}{\sigma^{2}}$	Measures the association of pairs of pixels under observation.

Cluster prominence: (cprom) [49–52]	$\overset{N_{g}}{\underset{i=1}{\sum }}\overset{N_{g}}{\underset{j=1}{\sum }}{\left(\left(i-\mu \right)+\left(j-\mu \right)\right)}^{4}p\left(i,j\right)$	Helps to measure the symmetry in the image matrix. If there is a high level of asymmetry in the canopy, it may be an indication of a shunted growth in the plant due to stress.

Cluster shade (cshad) [49–52]	$\overset{N_{g}}{\underset{i=1}{\sum }}\overset{N_{g}}{\underset{j=1}{\sum }}{\left(\left(i-\mu \right)+\left(j-\mu \right)\right)}^{3}p\left(i,j\right)$	Helps to measure skewness which is an indication of asymmetry. A high level of asymmetry implies that there is some problem in the growth of the plant due to which symmetrical canopy is not there.

Dissimilarity (Dissi) [49–52]	$\overset{N_{g}}{\underset{i=1}{\sum }}\overset{N_{g}}{\underset{j=1}{\sum }}\left|i-j\right|.p\left(i,j\right|$	Measure the mean difference between the pixels. It helps infer the level of similarity and homogeneity of the canopy structure.

Energy: (Energ) [49–52]	$\overset{N_{g}}{\underset{i=1}{\sum }}\overset{N_{g}}{\underset{j=1}{\sum }}p{\left(i,j\right)}^{2}$	Helps to measure the uniformity in the image matrix. There are no significant changes in the canopy morphology.

Entropy: (Entro) [49–52]	$-\overset{N_{g}}{\underset{i=1}{\sum }}\overset{N_{g}}{\underset{j=1}{\sum }}p\left(i,j\right)\log_{2}p\left(i,j\right)$	Helps to measure the degree of randomness. High randomness implies that there are a lot of changes occurring in the canopy structure.

Homogeneity: Homom (inverse difference moment) [49–52]	$\overset{N_{g}}{\underset{i=1}{\sum }}\overset{N_{g}}{\underset{j=1}{\sum }}\frac{1}{1+{\left(i-j\right)}^{2}}p\left(i,j\right)$	Measures homogeneity at the local level. There are not many changes occurring in the canopy due to any stimuli.

Homogeneity: (Homop) inverse difference [49–52]	$\overset{N_{g}}{\underset{i=1}{\sum }}\overset{N_{g}}{\underset{j=1}{\sum }}\frac{p\left(i,j\right)}{1+\left|i-j\right|}$	Measures the closeness of the pixels and the similarity between them.

Maximum probability (Maxpr) [49–52]	$\underset{i,j}{\mathrm{MAX}}p\left(i,j\right)$	Maxpr is a glcm metric that gives the max probability of finding pixels of interest for finding textures.

Sum of squares (sosvh): [49–52]	$\overset{N_{g}}{\underset{i=1}{\sum }}\overset{N_{g}}{\underset{j=1}{\sum }}{\left(1-\mu \right)}^{2}p\left(i,j\right)$	Helps to measure the mean/average shift between the pixels. High variance means there are a lot of changes occurring in the morphological structure.

Sum average (savgh) [49–52]	$\overset{2N_{g}}{\underset{k=2}{\sum }}p_{x+y}\left(k\right)k$	Helps to measure the average distribution of the gray levels. A high level of distribution of grey level means that the canopy is undergoing a high level of morphological changes.

Sum variance (svarh) [49–52]	$\overset{2N_{g}}{\underset{k=2}{\sum }}{\left(k-\mu_{x+y}\right)}^{2}p_{x+y}\left(k\right)$	Helps to measure pixel distributions in terms of dispersion.

Sum entropy (senth) [49–52]	$\overset{2N_{g}}{\underset{k=2}{\sum }}p_{x+y}\left(k\right)\log_{2}\left(p_{x+y}+\in \right)$	Helps to measures the disorder related to the gray level sum distribution of the image

Difference variance (Dvarh) [49–52]	$\overset{N_{g}-1}{\underset{k=0}{\sum }}{\left(k-\mu_{x-y}\right)}^{2}p_{x-y}\left(k\right)$	It helps to measure the heterogeneity level in the image. A low level of heterogeneity means images having similar patterns of pixels.

Difference entropy (Denth) [49–52]	$-\overset{N_{g}-1}{\underset{k=0}{\sum }}p_{x-y}\left(k\right)\log_{2}\left(p_{x-y}\left(k\right)+\in \right)$	It measures the disorder related to the gray level difference distribution of the image
Information measure of correlation1 (inf1h) [49–52]	$\frac{\text{HXY}-\text{HXY}1}{\max\left\{\text{HX},\text{HY}\right\}}$	Helps to measure the joint probability in terms of correlation and information it contains. A high value of information measure of correlation means the pixels are highly related to each other in terms of pattern.

Information measure of correlation2 (inf2h) [49–52]	$\sqrt{1-e^{-2\left(\text{HXY}2-\text{HXY}\right)}}$	Helps to measure dependency between the pixels. Higher dependency between pixels implies that if one of the pixels changes it will impact the other pixel and would bring significant changes in the texture of the canopy.

Inverse difference (INV) is homom (Homom.1) [49–52]	$\overset{N_{g}}{\underset{i=1}{\sum }}\overset{N_{g}}{\underset{j=1}{\sum }}\frac{p\left(i,j\right)}{1+\left|i-j\right|}$	Helps to measure local homogeneity. Indirectly help to monitor the canopy structure.

Inverse difference normalized (INN) [49–52]	$\overset{N_{g}}{\underset{i=1}{\sum }}\overset{N_{g}}{\underset{j=1}{\sum }}\frac{p\left(i,j\right)}{1+\left(\left|i-j\right|/N_{g}\right)}$	Helps to measure the difference between neighbouring pixel using normalized values.

Inverse difference moment normalized (idmnc) [49–52]	$\overset{N_{g}}{\underset{i=1}{\sum }}\overset{N_{g}}{\underset{j=1}{\sum }}\frac{p\left(i,j\right)}{1+\left({\left|i-j\right|}^{2}/N_{g}\right)}$	It computes the difference between the neighbouring intensity values that are normalized by the total number of discrete intensity values.

**Table 3:** Classification of glcm factors based on correlation ranges.

Correlation relationship	Correlation range	GLCM factors as per correlation
A (negative correlation)	-1 to 0.09	[“autocor,” “contr,” “ corrm,” “corrp,” “cprom,” “cshad,” “dissi,” “energ,” “entro,” “homom,” “homop,” “maxpr,” “sosvh,” “savgh,” “svarh,” “senth,” “dvarh,” “denth,” “inf1h,” “inf2h,” “homom.1,” “indnc,” “idmnc”]

B (low correlation)	0 to 0.49	[“autoc,” “contr,” “corrm,” “corrp,” “cprom,” “cshad,” “dissi,” “entro,” “sosvh,” “savgh,” “svarh,” “senth,” “dvarh,” “denth,” “inf1h,” “inf2h,” “ idmnc”]

C (medium correlation)	0.5 to 0.89	[“autoc,” “contr,” “cprom,” “cshad,” “dissi,” “energ,” “entro,” “homom,” “homop,” “maxpr,” “sosvh,” “savgh,” “svarh,” “senth,” “dvarh,” “denth,” “inf2h,” “homom1,” “indnc”]

D (high correlation)	0.9 to 1.00	[“autoc,” “contr,” “corrm,” “corrp,” “cprom,” “cshad,” “dissi,” “energ,” “entro,” “homom,” “homop,” “maxpr,” “sosvh,” “savgh,” “svarh,” “senth,” “dvarh,” “denth,” “homom.1,” “indnc”]

**Table 4:** Agglomeration clustering schedule.

Stage no.	Cluster combined		Coefficients	Stage cluster first appears		Next stage
	Cluster 1	Cluster 2		Cluster 1	Cluster 2	
1	2	17	.000	0	0	14
2	3	4	.000	0	0	20
3	9	23	.000	0	0	6
4	10	11	.367	0	0	10
5	8	12	.452	0	0	10
6	9	16	.849	3	0	9
7	1	15	1.358	0	0	8
8	1	13	1.493	7	0	12
9	9	18	1.820	6	0	17
10	8	10	3.854	5	4	16
11	5	6	9.366	0	0	15
12	1	14	9.856	8	0	15
13	21	22	24.732	0	0	16
14	2	7	27.952	1	0	18
15	1	5	32.500	12	11	17
16	8	21	58.100	10	13	21
17	1	9	62.709	15	9	18
18	1	2	90.114	17	14	19
19	1	20	257.481	18	0	20
20	1	3	443.493	19	2	22
21	8	19	2099.203	16	0	22
22	1	8	2152.905	20	21	0

**Table 5:** Comparative cluster membership analysis with different linkages.

S. no.	Indicator/variables	Between linkage	Centroid linkage	Furthest neighbour linkage	Median linkage	Nearest neighbour linkage	Ward's linkage	Within linkage
1	Autocorrelation (autoc)	1	1	1	1	1	1	1
2	Contrast (contr)	1	1	1	1	1	1	1
3	Correlation (corrm)	1	1	1	1	1	1	1
4	Correlation (corrp)	1	1	1	1	1	1	1
5	Cluster prominence (cprom)	1	1	1	1	1	1	1
6	Cluster shade (cshad)	1	1	1	1	1	1	1
7	Dissimilarity (dissi)	1	1	1	1	1	1	1
8	Energy (energ)	2	2	2	2	2	2	2
9	Entropy (entro)	1	1	1	1	1	1	1
10	Homogeneity (homom)	2	2	2	2	2	2	2
11	Homogeneity (homop)	2	2	2	2	2	2	2
12	Maximum probability (maxpr)	2	2	2	2	2	2	2
13	Sum of squares: variance (sosvh)	1	1	1	1	1	1	1
14	Sum average (savgh)	1	1	1	1	1	1	1
15	Sum variance (svarh)	1	1	1	1	1	1	1
16	Sum entropy (senth)	1	1	1	1	1	1	1
17	Difference variance (dvarh)	1	1	1	1	1	1	1
18	Difference entropy (denth)	1	1	1	1	1	1	1
19	Information measure of correlation1 (inf1h)	3	3	3	3	3	3	3
20	Information measure of correlation2 (inf2h)	3	3	3	3	3	3	3
21	Inverse difference (INV) is homom (homom1)	2	2	2	2	2	2	2
22	Inverse difference normalized (INN) (indnc)	2	2	2	2	2	2	2
23	Inverse difference moment normalized (idmnc)	1	1	1	1	1	1	1

## Data Availability

The data used to support the findings of this study are included within the article.

## Additional Points

*Future Perspective*. To overcome the limitations of this current work, a concept such as data fusion, data aggregation, and data augmentation may be utilized for deeper analysis and insights.
